# The transmission ecology of *Tahyna orthobunyavirus* in Austria as revealed by longitudinal mosquito sampling and blood meal analysis in floodplain habitats

**DOI:** 10.1186/s13071-021-05061-1

**Published:** 2021-10-30

**Authors:** Jeremy V. Camp, Edwin Kniha, Adelheid G. Obwaller, Julia Walochnik, Norbert Nowotny

**Affiliations:** 1 Viral Zoonoses, Emerging and Vector-Borne Infections Group, Institute of Virology, University of Veterinary Medicine Vienna, Vienna, Austria; 2 Institute of Specific Prophylaxis and Tropical Medicine, Center for Pathophysiology, Infectiology and Immunology, Medical University of Vienna, Vienna, Austria; 3 Division of Science, Research and Development, Federal Ministry of Defense, Vienna, Austria; 4 Department of Basic Medical Sciences, College of Medicine, Mohammed Bin Rashid University of Medicine and Health Sciences, Dubai, UAE; 5 Present Address: Center for Virology, Medical University of Vienna, Vienna, Austria

**Keywords:** *Orthobunyavirus*, Arbovirus, Mosquito, Transmission ecology, Blood meal analysis, Austria

## Abstract

**Background:**

*Tahyna orthobunyavirus* (TAHV) is a mosquito-borne virus that may cause mild flu-like symptoms or neurological symptoms in humans. It is historically associated with floodplain habitats in Central Europe, and the mammalophilic floodwater mosquito, *Aedes vexans*, is thought to be the principal vector. There are few contemporary reports of TAHV transmission ecology within mosquitoes or their vertebrate hosts, and virus infections are rarely reported (and probably seldom diagnosed). The objectives of this study were to survey the mosquito population for TAHV in three floodwater habitats and describe host usage by the predominant floodwater mosquito species to potentially define TAHV transmission at these foci.

**Methods:**

We performed longitudinal mosquito sampling along three major rivers in eastern Austria to characterize the mosquito community in floodplain habitats, and tested for the presence of TAHV in pools of mosquitoes. We characterized TAHV rescued from mosquito pool homogenate by sequencing. We surveyed mosquito host selection by analyzing mosquito blood meals.

**Results:**

We identified TAHV in two pools of *Ae. vexans* captured along the Leitha River. This mosquito, and other floodwater mosquitoes, used large mammals (red deer, roe deer, wild boar) as their hosts. The sequence of the rescued virus was remarkably similar to other TAHV isolates from the region, dating back to the first isolate of TAHV in 1958.

**Conclusions:**

In general, we confirmed that TAHV is most likely being transmitted by *Ae. vexans*, although the precise contribution of vertebrate-amplifying hosts to the ecological maintenance of the virus is unclear. The pattern of host selection matches the estimated exposure of the same large mammal species in the region to TAHV based on a recent serosurvey, but hares were also hosts at the site where TAHV was detected. We also confirm humans as hosts of two floodwater mosquito species, providing a potential mechanism for spillover of TAHV or other mosquito-borne viruses.

**Graphical Abstract:**

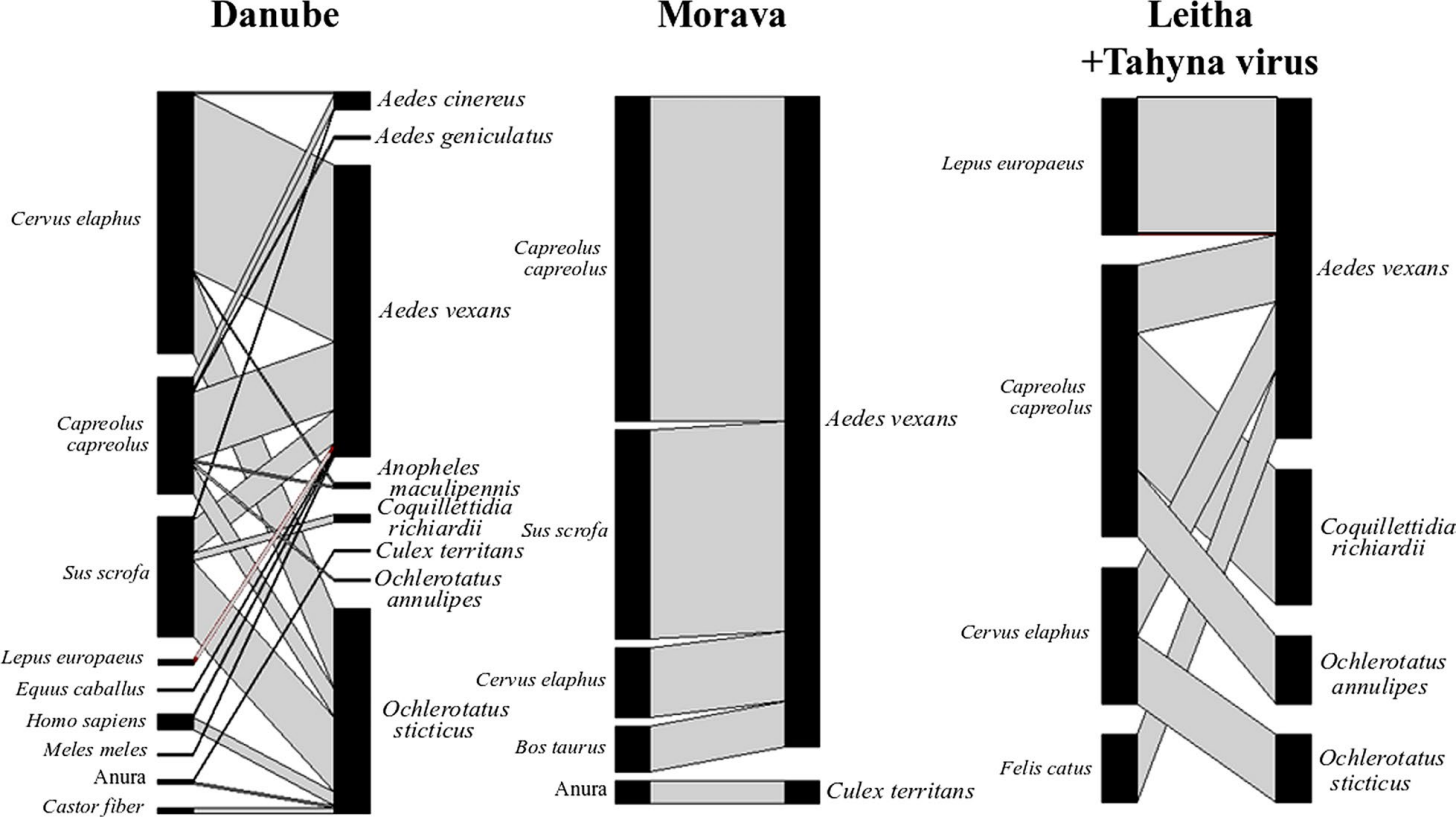

**Supplementary Information:**

The online version contains supplementary material available at 10.1186/s13071-021-05061-1.

## Background

*Tahyna orthobunyavirus* (TAHV) is a California encephalitis group virus in the family *Peribunyaviridae* (order *Bunyavirales*). TAHV was first isolated from mosquitoes near the village of Ťahyňa in eastern Slovakia in 1958 [1]. Soon after, additional isolations of TAHV from humans with acute influenza-like illness in the south Moravian region of the Czech Republic, near Valtice, led researchers and physicians to refer to the human disease caused by TAHV as “Valtice fever” [2–6]. Retrospective epidemiological studies in the Czech Republic and Russia suggested that TAHV causes a mild influenza-like disease in the majority of human cases, and may cause both acute and chronic neurological symptoms in a relatively large percentage of patients [4, 5, 7, 8]. Experimental animal infections (laboratory mice and rhesus macaques) have also supported neuroinvasive and occasionally neurovirulent phenotypes of TAHV [9, 10]. An in vitro comparison of a panel of field-derived isolates provided circumstantial evidence that disease phenotype may be associated with viral genotype [11]. Clinically, the virus shares similarities with another California group orthobunyavirus, *La Crosse orthobunyavirus*, in the United States, and shares some epidemiological properties related to their similar modes of transmission.

The virus has been isolated or detected in mosquitoes from many countries in Central and Eastern Europe (Additional file 1: Table S1), and serological evidence from animals and humans suggests the virus was widespread throughout continental Europe [12–23]. More recently, surveys from Austria and the Czech Republic suggest the virus is still circulating in the region [17, 22, 24, 25]. It has been isolated from mosquitoes in China with associated human case reports [26–28]. Reports of TAHV in Africa are based exclusively on serological studies [29–31] and are probably evidence of infections with the closely related Lumbo virus [32]. Although the virus has been detected in several species of mosquitoes, most of which are floodwater-associated aedine species (genus *Aedes* or *Ochlerotatus*), vector competence has been experimentally demonstrated only for *Aedes vexans* [33]. In Europe, increased seroprevalence in humans appears to be correlated with living close to rivers and with recent flooding events in floodplain habitats [20, 24, 34]. Human disease incidence is also correlated with age, with children more likely to be symptomatic and seroprevalence increasing to up to 80% in elderly populations, and with time of year, with the majority of cases in the late summer months [13, 19, 20, 24, 35–40].

In the two decades following the discovery of TAHV virus, researchers in the Czech Republic, Slovakia, and Austria focused intensely on defining the patterns of zoonotic transmission. A series of experimental infections of wild mammals provided evidence that most mammals endemic to the region support at least a transient viremia (e.g., [41–43]), and a series of serosurveys pointed to hares (*Lepus europaeus*) and possibly large mammals (wild boar, red deer, and domestic livestock) as having the highest exposure frequency [12, 18], reviewed in [14]. Some researchers focused on implicating the hedgehog (*Erinaceus europaeus*) as both a vertebrate host and an overwintering host [14, 44]. Although the involvement of hedgehogs in TAHV transmission was not clearly supported, hedgehogs are persistently listed as important vertebrate hosts [45–47].

Even though it is associated with neurovirulence, TAHV infection is not a notifiable disease. In fact, there are few, if any, reports of human infections in the last decades, and it is infrequently included in published virosurveys of mosquitoes. We recently performed a serosurvey of large mammals (red deer, roe deer, and wild boar) in Central Europe (Austria, Hungary, and Romania) and found that seroprevalence appeared to be unchanged compared to earlier reports: using wild boar in Austria as a reference, approximately 30% had virus-neutralizing antibodies in 1970 and 2019 [12, 17]. We also observed that seroprevalence varied by location. As this entire region has a similar climate (“Cfb,” temperate oceanic), we reasoned that TAHV transmission activity may vary in specific habitats due to the presence and/or relative abundance of specific competent vectors and competent vertebrate-amplifying hosts as has been demonstrated for other California encephalitis serogroup orthobunyaviruses [48]. In Austria, recent studies have demonstrated that mosquito assemblages vary according to landscape structure—specifically the distance to wetland sites and the land cover classification—and therefore factors such as habitat disturbance may influence the co-distribution of competent vectors and hosts [49]. Much remains unknown about the enzootic transmission, spillover risk, and current incidence of TAHV in humans. In the 60 years since its first isolation, additional tools have become available to investigate the transmission ecology of arboviruses—the most important of which are polymerase chain reaction (PCR)-based techniques to identify the hosts from the blood of resting mosquitoes and to more efficiently identify potential vectors in field surveys (i.e., highly sensitive quantitative reverse transcriptase PCR [RT-qPCR]).

We therefore undertook a multiyear survey of mosquitoes in several floodplain habitats in eastern Austria to investigate the transmission ecology of TAHV using contemporary techniques. Our approach was based on prior research from the 1960s–1970s, that the principal vector of TAHV is *Ae. vexans*, and small mammals act as amplifying hosts. Accordingly, we sampled floodplain habitats, describing the mosquito communities, host usage, and virus prevalence in each. As we previously noted that transmission activity (estimated by seroprevalence in large mammals) seemed to differ between locations, we sampled three separate floodplains to investigate whether we could identify correlates of virus presence and/or prevalence with aspects of the mosquito communities in each location, including relative species abundance and species diversity.

## Methods

### Study sites

**Fig. 1:**
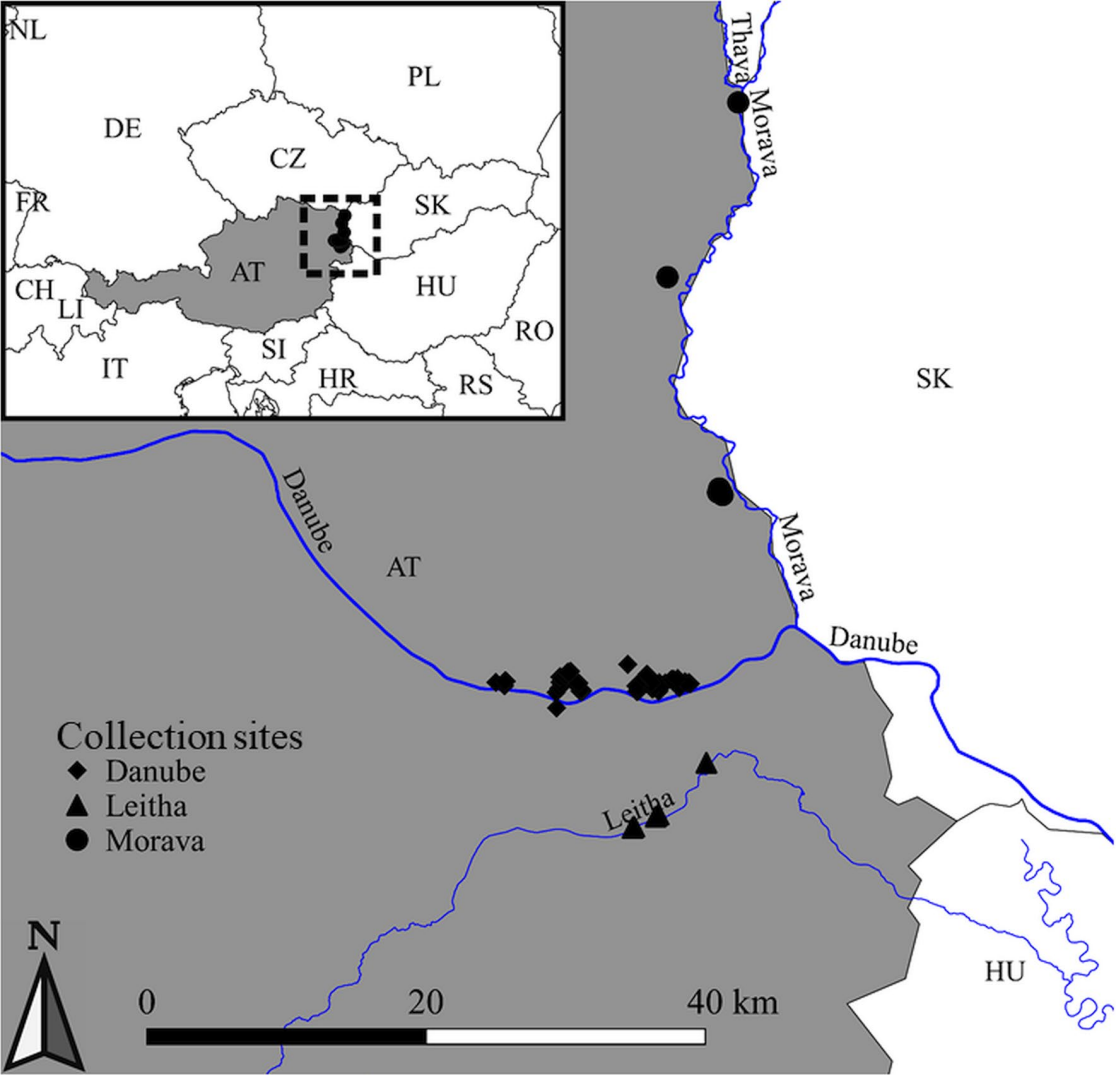
Map of eastern Austria showing collection sites (symbols) for mosquitoes along major rivers (blue lines). Symbols show sites along the Danube (diamonds), Leitha (triangles), and Morava (circles) rivers. Austria is shown in gray background, and its position relative to other countries in Europe is shown in the inset figure, where countries are marked with two-letter abbreviations (e.g., *AT* Austria, *SK* Slovakia, and *HU* Hungary are shown on the main map)

We selected floodplain habitats from three major waterways in eastern Austria: the Danube River, the Morava River, and the Leitha River (Fig. 1). Several locations were chosen along each river for longitudinal sampling (Additional file 1: Table S2). The Danube River is a historically managed waterway, with hydroelectric dams upriver from the study sites. Three collection sites below the levee along the Danube River within the National Park Donau-Auen were selected, as this is a large protected natural area and may occasionally flood. In contrast, the Morava River is largely an unmanaged waterway, although levees do exist to protect the surrounding farmlands. Although three sites were chosen initially, sampling along the Morava River was focused almost entirely within a World Wild Fund for Nature (WWF) conservation site near Marchegg in 2017, as mosquito control measures (application of granular Bti) are performed in areas upriver during summer months. The Leitha River is also a managed waterway, with canals and catchments to divert floodwaters, and thus seldom floods. We chose three sites along the Leitha in suburban habitats near the villages of Bruck an der Leitha and Rohrau, only two of which were sampled in 2017. Of the three floodplains, the Morava River is the least disturbed habitat, and the Leitha River is the most disturbed habitat.

### Mosquito collection

We performed weekly sampling of the adult questing mosquito population using Centers for Disease Control and Prevention (CDC) miniature light traps (John W. Hock Company) with fluorescent tube lighting. One kilogram of dry ice pellets was hung above the traps in an insulated canister with a plastic tube to direct the eluted CO_2_ towards the trap intake. Dry ice was used at all sites except for 2019 sampling along the Leitha, where only light traps with filament bulbs were used. Traps were started ﻿approximately 1 h before sunset, and the contents were collected the following morning no later than 1 h after sunrise. Resting mosquitoes were collected in the morning following trapping with a sweep net technique. Five 10-m-long transects were selected haphazardly around the light trap and a 50-cm diameter fine mesh insect net was continuously swept along the emergent or groundcover vegetation (mostly grasses) while walking the transect. The collections from each transect were aspirated into a cup with a backpack vacuum for transport. The collections from 2016 to 2017 were transferred to a laboratory at ambient temperature in a container lined with moistened paper towels, anesthetized for 1 min at −20 °C, and sorted on a frozen plate under a dissecting microscope. The collections from 2019 were stored at −20 °C and sorted later. Mosquitoes were identified according to a dichotomous key [50] (noting specific taxonomic issues in the Additional file 1). A variable size pooling strategy was used, pooling all unfed individuals (collected by light traps or by sweep nets) by species, collection date, and location in pool sizes of 10, 20, 30, 40, and 50 individuals, sequentially. Pools were stored at − 80 °C until processing. Blood-engorged females were not included in the pools, but were stored individually at − 80 °C.

While collection methods were standardized, sampling effort varied between years and study areas (Additional file 1: Table S3). The Danube study site was sampled over 23 trap-nights in 2016, and 11 trap-nights in 2017, from May until September in each year. The Morava study site was investigated once in 2016 (July) and regularly sampled over eight trap-nights from June to September in 2017. The Leitha study site was sampled over five trap-nights in 2017, from June to August, and over 34 trap-nights in 2019, from June to August (as noted, the 2019 sampling along the Leitha floodplain was performed using light traps without a source of CO_2_).

### Virus screening and isolation

Homogenization medium was made of Dulbecco’s modified Eagle medium (DMEM) supplemented with 20% bovine serum albumin, 1% penicillin/streptomycin, 10 µg/ml gentamicin, and 0.25 µg/ml amphotericin B (all from Gibco, Thermo Fischer Scientific). The homogenization medium was prepared fresh each day and kept on wet ice prior to adding to each mosquito pool in a volume according to the size of the pool (either 250 or 500 µl). Two to three metal beads were added to each pool, and the pools were homogenized on a TissueLyser bead mill (QIAGEN GmbH, Hilden, Germany) for 1 min shaking at 30 Hz. The homogenate was cleared by centrifugation in a 4 °C benchtop centrifuge for 20 min at 4000×*g*. The supernatant was removed, 200 µl were taken for RNA extraction, and the remaining supernatant and pellet were frozen at −80 °C separately. Total RNA was extracted from supernatant using a commercial kit (QIAamp viral RNA extraction kit) with a QIAcube high throughput liquid handling robot (both from QIAGEN), and eluted in 50 µl.

Pools were screened for viral RNA by RT-qPCR using a commercial kit (Luna Universal Probe One-Step RT-qPCR, New England Biolabs GmbH, Frankfurt, Germany) with primers targeting a portion of the S segment (TsF205: 5′CAGGTGGAGGTCGTCAATAAT; TsR291: 5′AGCACCCATCTAGCCAAATAC) and a fluorescent probe (TsP256: 5′-6FAM-ATAACAACGATCCTTACCATCCACCGGCTA-BHQ1) using 5 µl of homogenate supernatant RNA extract under the following cycling conditions: 55 °C for 10 min, 95 °C for 1 min, and 45 cycles at 95 °C for 10 s followed by a 1 min extension/acquisition step at 60 °C. An RNA extract of virus culture (TAHV strain Bardos-92, 10^6.5^ TCID50/ml) was included as a positive control. Putative positive pools were confirmed by conventional RT-PCR (One*Taq* One-Step RT-PCR, New England Biolabs) using primers targeting a 250-nucleotide (nt) region of the viral RNA (BCS82C and BSC332V) as described previously [51, 52].

### Virus sequencing

To isolate virus from the cleared homogenate of mosquito pools that were positive for viral RNA, Vero E6 cells (ATCC No. CRL-1586) were seeded onto a six-well plate in DMEM with 2% fetal calf serum and antibiotics and placed overnight at 37 °C in a humidified incubator. The medium was removed and 100 µl diluted homogenate (1:0, 1:10, and 1:100) was added to each of three wells. After 1 h incubation at 37 °C with rocking, the inoculum was removed and fresh medium was added. The plates were incubated at 37 °C for up to 6 days, inspecting for cytopathic effect each day. Upon detection of cytopathic effect, cell culture medium was removed and centrifuged to remove cells and cellular debris. Total RNA was extracted using a commercial kit (Quick-RNA, Zymo Research, Irvine, CA, USA), and the presence of TAHV viral RNA was tested as described above. When viral RNA was detected, the near-complete genome was amplified by conventional RT-PCR using a panel of primers designed to cover all but the conserved 3′ and 5′ ends (Additional file 1: Table S4) [11, 53, 54]. The amplicons were sequenced by the Sanger method, primer regions were removed, and sequences were aligned to the reference sequence (the prototype TAHV isolate *Aedes caspius*/Slovakia/Bardos92/1958, GenBank accession numbers HM036208, HM036212, and HM036210 for the S, M, and L segments, respectively) using the Muscle algorithm in MEGA v. 7 [55].

The resulting consensus sequences for the coding regions of the nucleocapsid protein (“NP,” S segment, 705 nt without the final stop codon), glycoprotein polyprotein precursor (“G1/G2,” M segment, 4320 nt), and RNA-dependent RNA polymerase (“RdRP,” L segment, 6789 nt) were compared to previously published sequences (Additional file 1: Table S5) with a phylogenetic analysis using maximum likelihood methods. The optimal substitution models for each gene were determined by “SMS” [56], comparing the Akaike information criterion (AIC), to be GTR + I for NP, GTR + G + I with four gamma categories for the G1/G2, and GTR + G with four gamma categories for the RdRP. The phylogenies were inferred over 500 bootstrap samplings.

### Blood meal analysis

Blood-engorged females were collected in sweep nets or occasionally in light traps, and processed to identify their vertebrate host. The abdomens were separated from individual frozen mosquitoes and crushed with a pestle in 50 µl sterile buffered saline (Gibco). A commercial kit (DNeasy) was used to extract DNA from the blood meal suspension. Amplicons from a PCR protocol targeting vertebrate 16S rRNA were sequenced by the Sanger method (primers L2513 and H2714 [57]), and sequences were compared to the National Center for Biotechnology Information (NCBI) database using a BLASTn search. Blood meal-derived sequences matching references with > 99% identity were considered a positive identification, and an in-house voucher DNA database from most hosts (all native amphibians and reptiles, many native mammals, and some birds) was available to confirm identification. Multiple meals were detected by inspecting the sequencing chromatograph for overlapping peaks at specific sites. As this primer set may have a bias towards amplifying some vertebrate hosts and not others, amplicon-negative samples were subjected to two additional PCR amplification protocols targeting vertebrate cytochrome b [58, 59] or cytochrome oxidase I [60], both using nested PCR as described therein. For the first-round PCR protocols, we used 2 µl DNA template in a 25 µl total reaction mixture that included Go*Taq* G2 polymerase (New England Biolabs), 0.5 µM primer mixes, and 0.2 mM dNTPs. Nested reactions included 0.5 µl products from the first-round PCR in 50 µl reaction mixtures. Cycling conditions and annealing temperatures were followed exactly as described in the originally published protocols [57–60].

### Statistical analysis

To describe the mosquito communities and the hosts used by each species, species richness (*S*_*boot*_) was estimated by a bootstrap method pooled over trap-nights (“specpool” in R package vegan). For similar descriptions, species diversity was estimated by calculating Shannon’s entropy as $H^{\prime} = - \sum p_{i} \ln p_{i}$, where *p*_*i*_ is the proportional abundance of a single species, and Pielou’s evenness was calculated as *J′* = *H′* / ln *S*, where *S* is the observed species richness. Mosquito–host interactions were visualized with bipartite plots (“plotweb” in R package bipartite).

As sampling effort varied between floodplains and between years, we compared between floodplains using adjusted abundance (abundance per trap-night). To test whether there were floodplain-specific differences in species (adjusted) abundance, we first tested whether there was conditional independence between floodplain and species using a Chi-square test. As a post hoc test, to describe specific differences, standardized Pearson residuals were calculated and expected values were based on the joint probability between adjusted abundance per species and adjusted abundance per floodplain [61]. Standardized residuals greater than 2 or less than −2 were considered significant, and species-specific differences in adjusted abundance between floodplains were inferred when significantly more and fewer were captured than expected in different floodplains, respectively. A similar statistical approach was used to test species-specific differences in host usage for a subset of hosts (i.e., conditional independence between mosquito species and host usage) using raw counts and not adjusted abundances. We compared the minimum virus-positive ratio between sites for *Ae. vexans* using Fisher’s exact tests, assuming that only one mosquito was virus-positive in virus-positive pools.

## Results

### Floodwater mosquito assemblages

In total, 23,748 mosquitoes were captured over 84 trap-nights (Table 1). The predominant mosquito species were typical floodwater mosquitoes, with *Ae. vexans* comprising 65% of the total collections, and *Ochlerotatus sticticus* comprising 20% of the total collections. In particular, *Ae. vexans* made up 95% of the total captures along the Morava River. Overall, there were significant differences between species adjusted abundance and floodplain (*χ*^2^ = 1824, *df* = 28, *P* < 0.0001). There were significantly more *Coquillettidia richiardii* and *Oc. sticticus* captured along the Danube than expected. Both *Anopheles maculipennis* sensu lato and *Culex pipiens* s.l. were more abundant along the Leitha River than the other locations. There were other floodplain-specific species associations, wherein less abundant species (< 1% of the total collection) were present in some but absent from other habitats (Table 1). Most notably, *Ochlerotatus cantans*, another floodwater species, was not captured along the Danube River but was present in the collections from both the Morava and Leitha rivers.

**Table 1:** Mosquito abundance and biodiversity estimates (*S* = observed species richness, *S*_boot_ = bootstrapped estimated richness with standard error [SE], *H′* = Shannon diversity index, and *J′* = Pielou’s species evenness) from three floodplains in eastern Austria, 2016–2019, with species sorted by total abundance (^a^floodwater aedine species)

Species	Danube	Morava	Leitha	Total
*Aedes vexans*^a^	4557	9889	1243	15,689
*Ochlerotatus sticticus*^a^	4755	51	123	4929
*Coquillettidia richiardii*	1229	90	0	1319
*Anopheles maculipennis* s.l.	218	52	384	654
*Aedes cinereus*^a^	427	73	13	513
*Ochlerotatus cantans*^a^	0	194	60	254
*Culex pipiens* s.l.	117	18	133	268
*Culex modestus*	2	30	3	35
*Anopheles plumbeus*	33	0	0	33
*Aedes geniculatus*	9	0	10	19
*Anopheles hyrcanus*	9	0	5	14
*Culiseta annulata*	4	0	9	13
*Culex territans*	4	0	0	4
*Anopheles claviger*	3	0	0	3
*Culiseta longiareolata*	1	0	0	1
Total	11,368	10,397	1983	23,748
Trap-nights	34	11	39	84
*S*	14	8	10	15
*S*_boot_ (SE)	15.0 (1.0)	8.0 (0.2)	10.5 (0.6)	15.6 (0.8)
*H′*	1.25	0.28	1.18	1.14
*J′*	0.47	0.14	0.51	0.42

**Fig. 2:**
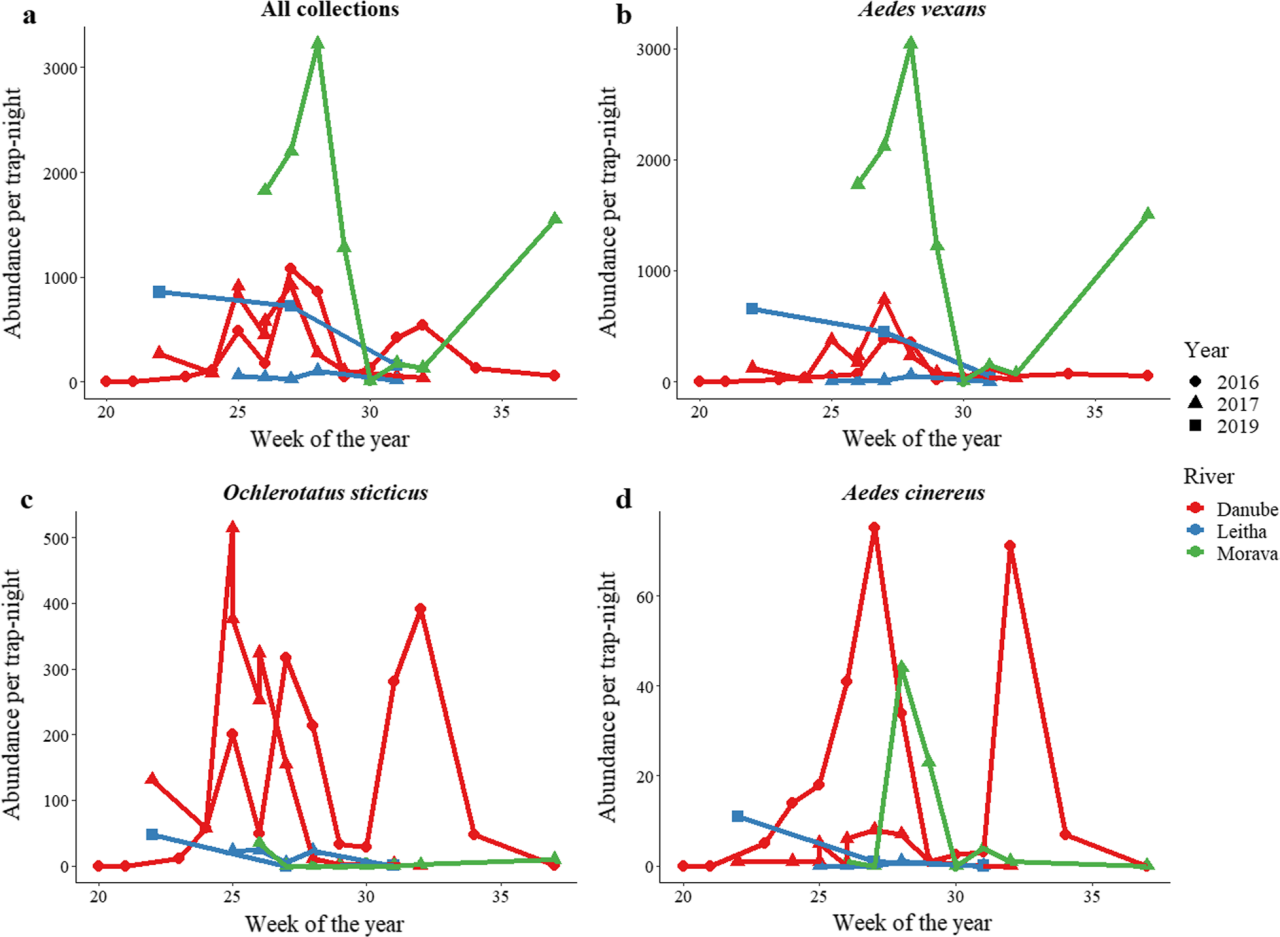
Mosquito abundance per trap-night in 3 years along three floodplains in eastern Austria, showing **a** all species and the three most abundant floodwater aedine species: **b** *Aedes vexans*, **c** *Ochlerotatus sticticus*, and **d** *Aedes cinereus*. The symbols mark the abundance per trap-night organized by the numeric week of each year: 2016 (circles), 2017 (triangles), and 2019 (squares). Trapping was performed from the middle of May (calendar week 20) until the middle of September (calendar week 37). The lines connect trap-nights and are colored by floodplain: Danube (red), Leitha (blue), Morava (green)

The phenology of the floodwater-associated aedine species showed a typical multivoltine pattern of abundance (Fig. 2). The peak abundances of *Ae. vexans* and *Oc. sticticus* along the Danube occurred 3–4 weeks after flooding events (inferred from maximum weekly water levels of the Danube), with the emergence of questing *Ae. vexans* females preceding *Oc. sticticus* by approximately 1 week (Additional file 1: Figure S1). Abundance per trap-night was highest along the Morava River, where collections were 95% *Ae. vexans*. In general, abundance was similar each year (Fig. 2); however, *Ae. vexans* was more abundant along the Leitha River in 2019 compared to 2017 and *Aedes cinereus* had increased abundance along the Danube in 2016 compared to 2017 (Fig. 2).

Although sampling effort was not equal, pooled species richness estimates suggested that all species were accounted for, possibly missing single species from both the Danube (*S* = 14, *S*_*boot*_ = 15) and Leitha rivers (*S* = 10, *S*_*boot*_ = 10.5) (Table 1). The species diversity was highest along the Danube (*H′* = 1.25) and Leitha (*H′* = 1.18) rivers, and considerably lower (*H′* = 0.28) along the Morava. The low calculated diversity was due to the large number of *Ae. vexans* captured along the Morava, thus making the evenness also comparatively lower for this site (*J′* = 0.14) compared to the Danube and Leitha (*J′* = 0.47 and 0.51, respectively). The mosquito communities in each habitat were enriched for aedine floodwater species (*Ae. vexans*, *Ae. cinereus*, *Oc. sticticus*, and *Oc. cantans*) with 86, 98, and 72% of total collections along the Danube, Morava, and Leitha, respectively (Table 1). Species whose immatures are associated with permanent and semi-permanent standing water, including peridomestic species such as *Cx. pipiens* s.l., were highest in the more disturbed habitats along the Leitha (27% of all captures), and species which mature in tree holes (*Aedes geniculatus* and *Anopheles plumbeus*) were less than 1% of captures at each site (not captured along the Morava).

### Identification of Tahyna virus in mosquitoes

In total, 824 pools were screened for TAHV by RT-qPCR, including 451 pools of *Ae. vexans* (Table 2). Viral RNA was detected in two pools of *Ae. vexans* collected in 2019 from a trap site on the Leitha River. The calculated minimum virus-positive ratio in *Ae. vexans* at the Leitha River (2/1243) was statistically higher than at the Danube (0/4557) and the Morava (0/9889) by Fisher’s exact test (*P* = 0.046 and 0.012, respectively). The cycle threshold values from the RT-qPCR tests were 15.7 and 23.6 for pools OadD806 and OadD823, respectively, and the positive control (a 10^6.5^ TCID50/ml virus culture) was 19.2. The cleared homogenates from these two pools were placed on Vero cells and a cytopathic effect was seen by 3 days post-infection in all three dilutions of the pool homogenate (1:10, 1:100 and 1:1000) demonstrating an effective concentration of at least 10^4^ TCID/ml. The cleared cell culture supernatant was positive for TAHV, and a primer walking strategy was used to amplify and sequence the coding-complete genome of TAHV of both isolates: Austria/OadD806/2019 (Genbank accession numbers S: MZ245724, M: MZ245726, L: MZ245728) and Austria/OadD823/2019 (S: MZ245725, M: MZ245727, L: MZ245729). However, the complete sequence of the L segment from isolate Austria/OadD806/2019 was not obtained (missing nucleotide positions 2357–3215).

**Table 2:** Mosquito pools (and total individuals) screened for TAHV by RT-qPCR by year, floodplain, and mosquito species

Mosquito	Danube		Morava		Leitha		Total
	2016	2017	2016	2017	2017	2019	
*Ae. vexans*	61 (2129)	84 (2428)	1 (5)	276 (9884)	6 (85)	23 (1158)^a^	451 (15,689)
*Oc. sticticus*	71 (2927)	52 (1828)	1 (4)	3 (47)	4 (75)	2 (48)	133 (4929)
Others	129 (2139)	36 (241)	0	38 (511)	18 (87)	19 (530)	240 (3508)
Total^b^	261 (7195)	172 (4497)	2 (9)	317 (10,442)	28 (247)	44 (1736)	824 (24,126)

^a^Two pools were positive for TAHV nucleic acids

^b^The differences in total counts compared to Table 1 are due to damaged specimens that were not identifiable to species but were nonetheless tested for virus

The coding sequences from each segment were compared to reference sequences of TAHV (Additional file 1: Table S5). We observed high similarity between the two isolates identified here and previously published TAHV sequences from Europe. The NP coding region of the S segment was the most conserved gene analyzed, with greater than 99% pairwise nucleotide identity to other TAHV isolates from Europe, and 93–97% identity to isolates from China. There were no non-synonymous substitutions in the deduced amino acid sequence for the NP between the two sequenced isolates, and a single amino acid substitution was detected in the TAHV prototype sequence compared to other sequences from Europe (Table 3). The nucleotide sequences for G1/G2 and RdRP were also highly similar to other isolates from Europe, with 4–17 non-synonymous substitutions and relatively high (97.9–99.4%) pairwise nucleotide identities (*N.B.* the unsequenced region of OadD806 [MZ245728] was removed from other sequences for analysis).

**Table 3:** Summary statistics comparing the sequences of two TAHV isolates from Austria, 2019, to historical TAHV isolates from Europe (Czech Republic, Slovakia, and France; 1958–1984)

	OadD806	OadD823
NP^a^—% nt similarity^d^	99.3–99.8	99.1–99.7
NP—# aa substitutions^e^	0–1	0–1
G1/G2^b^—% nt similarity	98.0–99.3	97.9–99.4
G1/G2—# aa substitutions	4–17	4–15
RdRP^c^—% nt similarity	nd	98.2–99.3
RdRP—# aa substitutions	nd	5–11

Pairwise sequence comparisons for the coding regions for (a) nucleocapsid protein [“NP”]; (b) polyprotein precursor for G1 and G2 glycoproteins [“G1/G2”]; (c) RNA-dependent RNA polymerase [“RdRP”]. Numbers represent the ranges of (d) percent pairwise nucleotide [“nt”] similarities and (e) number of amino acid [“aa”] substitutions

Among the amino acid substitutions that were different between the two isolates, seven in G1/G2 and eight in the RdRP, only seven total substitutions were unique (Additional file 1: Table S6). Specifically, Austria/OadD23/2019 shared three non-synonymous mutations with two other historical isolates (Czech Republic/4057/1966 and Czech Republic/5060/1968) in G1/G2 with one additional “unique” substitution not found in other isolates, while Austria/OadD806/2019 had two unique substitutions and shared one substitution with another isolate. In the RdRP, the two isolates from Austria differed at site 2191, which is variable between all sequences (eight sequences from Europe have isoleucine and seven have valine at this position). Also in the RdRP, each isolate from Austria had two unique substitutions, while Austria/OadD806/2019 shared one substitution with three other isolates and Austria/OadD823/2019 shared two substitutions with one and three other isolates, respectively.

**Fig. 3:**
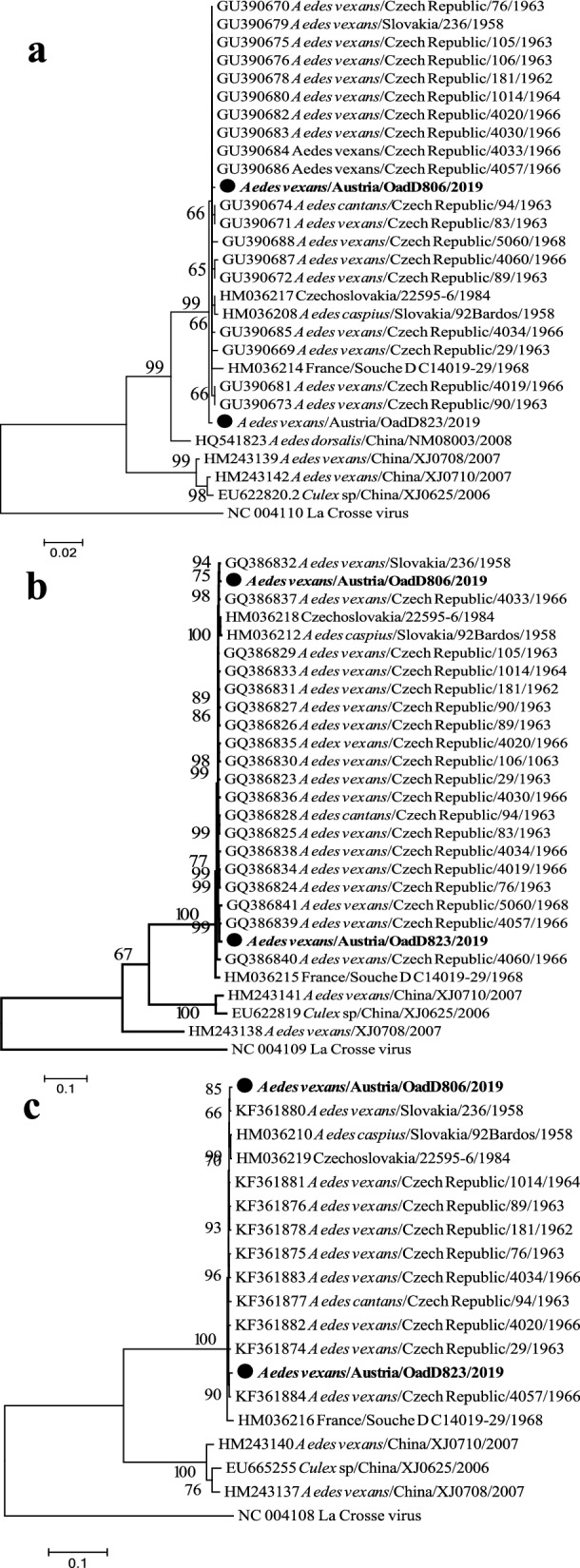
Maximum likelihood phylogenetic trees of the coding regions for *Tahyna orthobunyavirus* nucleocapsid protein (**a**), polyprotein (**b**), and partial RNA-dependent RNA polymerase (**c**) isolated from pools of mosquitoes. Two isolates from Austria (in bold text marked with black circles) are compared to historical isolates from Europe and China, and the reference sequence of *La Crosse orthobunyavirus* was used as an outgroup. Terminal branch names list the GenBank accession number, the species of mosquito, the country, the isolate number, and the year when known. The accession numbers for the two isolates from Austria are Austria/OadD806/2019 (S: MZ245724, M: MZ245726, L: MZ245728) and Austria/OadD823/2019 (S: MZ245725, M: MZ245727, L: MZ245729). The trees are inferred over 500 bootstraps using the GTR + I (**a**), GTR + G + I (**b**), and GTR + G (**c**) substitution models. The lengths of the branches in substitutions per site are indicated by the scale bar. Because 858 nucleotides were not sequenced from isolate Austria/OadD806/2019 (MZ245728, nucleotide positions 2357–3215), this region was removed from the other sequences for analysis, maintaining codons in-frame

Phylogenetic trees were built using full-length coding regions of TAHV isolates from Europe and China (Additional file 1: Table S5) with the reference sequence for *La Crosse orthobunyavirus* as an outgroup (GenBank accession numbers S: NC_004110, M: NC_004109, L: NC_004108). The 24 isolates from Europe included two from this study (Austria, 2019), 18 from the Czech Republic (1962–1966), two from Slovakia (1958), one from “Czechoslovakia” (1984, precise location unknown), and one from France (1968)—20 of the isolates were obtained from pools of *Ae. vexans*. The phylogenetic analysis shows the high similarity of the isolates from Europe, all of which shared a common ancestral node with four isolates from China (Fig. 3). Although it was not well supported, in each tree, sequences from the Czech Republic, Slovakia, and Austria seemed to share a common ancestor with the isolate from France (“France/Souche D C14019-29/1968”), possibly suggesting an isolation-by-distance pattern of genetic diversity. Notably, there was no apparent temporal grouping of any isolates from Europe in the phylogram.

### Blood meal analysis

The hosts were determined for 215 individual mosquitoes out of 273 captured blood-fed individuals (success rate of 79%, Additional file 1: Table S7) by genetic identification of blood in their guts. According to the sampling design, most of the resting, blood-engorged mosquitoes captured were the abundant floodwater aedine species: *Ae. vexans* (*N* = 141), *Oc. sticticus* (82), *Ae. cinereus* (12), and *Oc. annulipes* (2). Other species were also captured at low frequency, such as *Cq. richiardii* (6), *Cx. territans* (3), and *An. maculipennis* s.l. (4). With the exception of three individuals feeding on anurans (two *Cx. territans* and one *Oc. sticticus*), all hosts were identified as mammals (Table 4). One anuran host of *Cx. territans* was identified as a European tree frog (*Hyla arborea*), and the other two anuran hosts were identified as native hybridogenic *Pelophylax* spp. but we could not differentiate between the species using the sequenced region (Additional file 1: Table S7). The most common mammalian hosts were red deer (*Cervus elaphus*, *N* = 94), European roe deer (*Capreolus capreolus*, *N* = 55), and wild boar (*Sus scrofa*, *N* = 51). The seven other mammalian species identified as hosts included humans (*Homo sapiens*, 12), hares (*L. europaeus*, 4), cows (*Bos taurus*, 2), beavers (*Castor fiber*, 2), a domestic cat (*Felis catus*, 1), a horse (*Equus caballus*, 1), and a badger (*Meles meles*, 1) (Additional file 1: Table S7). We detected six mixed blood meals by inspection of the Sanger sequencing spectrographs from two of each of the following species: *Ae. vexans* (two mixed meals of wild boar and roe deer), *Oc. sticticus* (one mixed meal of red deer and roe deer, and one mixed meal of red deer and human), and *Ae. cinereus* (one mixed wild boar and roe deer, one mixed red deer and roe deer) (Table 4).

**Table 4:** Mammalian hosts of floodwater mosquitoes identified by blood meal analysis

Host	*Ae. vexans*	*Oc. sticticus*	Total^a^
*Cervus elaphus*	62	30^c,d^	92
*Capreolus capreolus*	36^b^	10^c^	46
*Sus scrofa*	20^b^	27	47
*Homo sapiens*	1	5^d^	6
*Lepus europaeus*	4	0	4
*Bos taurus*	2	0	2
*Castor fiber*	0	2	2
*Equus caballus*	1	0	1
*Felis catus*	1	0	1
*Meles meles*	1	0	1
Total^a^	128	74	202
*S*	9	6	11
*S*_boot_ (SE)	10.6 (1.0)	6.8 (0.7)	13.2 (1.3)
*H′*	1.32	1.28	1.39
*J′*	0.60	0.72	0.77

^a^Totals exclude 12 blood meals from five additional mosquito species

^b^Includes two mixed blood meals

^c^Includes one mixed blood meal

^d^Includes one mixed blood meal

As our analysis focused on the most abundant aedine floodwater species, *Ae. vexans*, and *Oc. sticticus*, we otherwise note that we identified two *C. capreolus* and three *S. scrofa* blood meals in *Cq. richiardii*; one *C. capreolus* blood meal in *Oc. annulipes*; and in *An. maculipennis* s.l. one each *C. elaphus* and *C. capreolus* blood meals (Additional file 1: Figure S2; Table S7)*.*

We estimated species richness (*S*) for each of the two most abundant floodwater species using a bootstrap approach over trap-nights to build the accumulation curves (Table 4). In general, sampling underestimated host species richness and *Ae. vexans* (estimated *S*_*boot*_ = 10.6) appeared to have a wider host range than *Oc. sticticus* (*S*_*boot*_ = 6.8). The calculated host species diversity was similar for both *Ae. vexans* and *Oc. sticticus* (*H′* = 1.32 and 1.28, respectively), although the evenness was higher for *Oc. sticticus* (*J′* = 0.60 and 0.72 for *Ae. vexans* and *Oc. sticticus*, respectively). Each of these two mosquito species took blood meals mostly from three large wild mammals (red deer, roe deer, and wild boar), and blood meals from six additional species were identified in *Ae. vexans*, whereas only two additional species were identified in *Oc. sticticus* (Table 4). Notably, two blood meals from *C. fiber* were observed in *Oc. sticticus* and none in *Ae. vexans,* whereas hares, cows, a badger, a horse, and a cat were identified in *Ae. vexans* but not in *Oc. sticticus* (Table 4). Focusing on the three most common host species in the blood meals of these two species, we found that *Ae. vexans* fed more often on roe deer and *Oc. sticticus* fed more often on wild boar than expected (χ^2^ = 13.9, *df* = 2, *P* < 0.0001; standardized residuals >|2| were considered significantly different than expected).

As we isolated TAHV from two pools of *Ae. vexans* at one field site, along the Leitha River, we were interested in analyzing differences in host selection between the three major floodplain habitats for this species. Although light trap sampling effort was similar between the Danube and Leitha sites (Additional file 1: Table S3), more blood meals were collected and identified from the Danube (Table 5). Due to this low sample size at the floodplain of interest—the Leitha River—we could not perform a robust statistical comparison between sites. Seven host species were identified in *Ae. vexans* blood meals from the Danube, four from the Morava, and three from the Leitha floodplains (Table 5). Over half of the blood meals from the Danube floodplain were from *C. elaphus*, half of the blood meals along the Morava floodplain were from *C. capreolus*, and two of the four blood meals from the Leitha floodplain were from *L. europaeus*, (Table 5).

**Table 5:** Hosts of *Aedes vexans* identified by blood meal analysis captured along three floodplain habitats in eastern Austria, 2016–2019

Host	Danube	Morava	Leitha	Total
*Cervus elaphus*	59	3	0	62
*Capreolus capreolus*	21^a^	14	1	36
*Sus scrofa*	11^a^	9	0	20
*Lepus europaeus*	2	0	2	4
*Bos taurus*	0	2	0	2
*Equus caballus*	1	0	0	1
*Felis catus*	0	0	1	1
*Homo sapiens*	1	0	0	1
*Meles meles*	1	0	0	1
Total	96^a^	28	4	128

^a^Includes two mixed meals

## Discussion

We provide a contemporary assessment of TAHV transmission ecology with a focus on virus–vector and host–vector interactions. Previous reports from the Czech Republic suggested that floodplain habitats in the southern region (South Moravia) near the confluence of the Morava and Thaya rivers were a focus of intense TAHV transmission and spillover to humans [62]. In Austria, researchers compared sites in two regions, focusing on seroconversion in wild mammals and detection of virus in pools of mosquitoes, and found that TAHV transmission was not focused in the Danube floodplain habitat, but rather in the Pannonian Basin, approximately 15 km south of where we identified TAHV in our study [12, 63]. Our previous sampling of the Pannonian Basin (in roughly the same location as the 1965 survey by Aspöck and Kunz [63]) yielded very few floodwater mosquito species, although we did not test for TAHV in those pools [64]. A recent survey in the mountainous western Austria identified TAHV in *Culex* spp., with very little seropositivity in humans [22]. Based on the presence of virus in *Ae. vexans* in our study, our data support the previous findings regarding the distribution of TAHV in eastern Austria, indicating that TAHV activity is low along the Morava and Danube rivers, but is higher in more southern regions along the Leitha River and possibly further south.

Collectively, the historical data suggest that TAHV is associated with floodwater habitats in eastern Austria and the southern Czech Republic, but may be focal in its distribution [17]. We therefore focused on three floodplains, comparing mosquito community composition, mosquito species relative abundance, and host usage, and were interested in correlating these aspects with differences in TAHV presence/prevalence. In general, all habitats had similar mosquito community composition: as expected, they were composed of *Ae. vexans* and other floodwater mosquitoes (*Oc. sticticus*, *Ae. cinereus*, and *Oc. cantans*) (Table 1) (cf. [65, 66]). In terms of relative species abundance, diversity, and evenness indices, the sites along the Leitha and the Danube rivers were very similar (Table 1); whereas collections along the Morava River were dominated by *Ae. vexans* with few other species being collected. Thus, there was no clear association of mosquito community composition with the presence/abundance of TAHV, only that periurban mosquito species (i.e., *Cx. pipiens* s.l.) were found in relatively higher abundances in the disturbed habitats along the Leitha, near where TAHV-positive *Ae. vexans* were discovered (Table 1). Otherwise, we believe that our sampling was comprehensive (e.g., species richness estimates in Table 1) and adequately described the dynamics of these mosquito communities (Additional file 1: Figure S1).

We noted that the floodplain along the Leitha River, where TAHV-positive mosquito pools were detected, had a much lower trapping success (mosquitoes per trap-night) than the other floodplains (Table 1). This was probably related to the difference in trapping method for questing mosquitoes at this site during 2019, as the light traps did not use a source of CO_2_, and may not reflect a lower abundance of mosquitoes. At least two in 1243 (0.16%) *Ae. vexans* captured along the Leitha River were positive for TAHV, and this was a statistically higher minimum virus-positive ratio compared to other floodplains.

The previous virosurveys of mosquitoes in Austria concluded that *Ae. vexans* was the principal vector, based on the greatest number of virus-positive pools [63, 67], and this seems to be the case throughout Europe (Additional file 1: Table S1). While we could detect viral RNA and isolate TAHV from two pools of *Ae. vexans*, this is not proof of vector competence. The proof of the vector competence of *Ae. vexans* was shown experimentally in the 1960s, exposing *Ae. vexans* to virus by feeding on infected laboratory rabbits, demonstrating their infection, then demonstrating transmission to naïve suckling mice [68, 69]. In addition, Rödl et al. demonstrated laboratory infection and transmission of *Ae. vexans* using wild-caught hares, which are presumed to be the most competent vertebrate-amplifying host [33]. Using a similar approach, Danielová et al. provided wild-caught mosquitoes blood from viremic laboratory rabbits and demonstrated vector competence for several other species (e.g., *Oc. sticticus*, and *Cs. annulata* but not in *Cx. pipiens* or *An. maculipennis*) [70, 71]. As the authors noted then, and as we note now, some of these species (e.g., *Cs. annulata*) are not in abundance in floodplain habitats in Austria and the Czech Republic and may not be as important as *Ae. vexans* in transmission and maintenance of TAHV in these geographic regions. In the Czech Republic, virus has been isolated from *Cs. annulata* larvae collected in the early spring [72], potentially implicating the species as an overwintering host. It is also interesting that *Oc. sticticus* was found to be a competent vector, and is in high abundance in floodplain habitats, but TAHV (to our knowledge) has rarely been isolated/detected in wild-caught specimens (Additional file 1: Table S1).

While many endemic wild vertebrate hosts were shown to become viremic in experimental infections with TAHV, it was unclear whether they were competent vertebrate hosts. As a result, there is some disagreement regarding the roles of mammal species in virus amplification in the literature [12, 14, 44]. We approached this question by combining a virus survey with a blood meal survey, to attempt to correlate host feeding with virus transmission indirectly. Although circumstantial (and not an indication of host competence), this approach has the benefit of measuring temporal and spatial associations between virus-positive vectors and their hosts in a given area (e.g., West Nile virus, St. Louis encephalitis virus, eastern equine encephalitis virus [73–75]). A principal limitation of our study is that we were unable to provide strong evidence of association between vectors and hosts when virus-positive mosquitoes were detected. However, we add to the growing literature on mosquito host-feeding strategies, highlighting the associations between native mosquito species in floodplain habitats in Europe.

We found that floodwater mosquitoes, particularly *Ae. vexans* and *Oc. sticticus*, primarily used large mammals as hosts: red deer (*C. elaphus*), roe deer (*C. capreolus*), and wild boar (*S. scrofa*). Others have reported a more catholic feeding behavior of *Ae. vexans*, in that avian hosts may occasionally be selected [58, 76–79], although we only identified mammals as hosts at the study sites here. We acknowledge that our blood meal analysis was biased, as we only performed sweep-net sampling of vegetation and likely limited the survey to mosquitoes feeding on hosts found in the immediate vicinity of our questing traps. In addition to the 220 hosts identified from blood meals, 58 individual mosquitoes appeared to have recently fed (28 of which were *Cx. pipiens* s.l.) but were amplicon-negative for the blood meal identification methods described (Additional file 1: Table S7). Blood meals that were amplicon-negative were analyzed by additional PCR assays to target other gene regions but these either amplified mosquito DNA or remained negative. Therefore, we cannot rule out the possibility that we failed to amplify avian blood meals, and acknowledge that our sampling technique was biased towards collecting mammal-feeding individuals. Although it is clear that birds are exposed to TAHV in floodwater habitats, prior research suggests it is unlikely that birds or ectothermic vertebrates are involved in the transmission ecology of TAHV [14, 15, 80, 81].

In addition to laboratory competence experiments, others have concluded that hares are the primary amplifying host of TAHV based on comparative seroprevalence to other wild animals in the Czech Republic and Austria as well as the coincidental timing of the emergence of virus-positive mosquitoes and seroconversion in wild hares and sentinel rabbits [12, 14, 62, 67, 68, 82, 83]. There has been no recent serosurvey of small mammal populations in Austria for TAHV-reactive antibodies; our recent survey included only red deer, wild boar, and roe deer [35]. We therefore find it important, although circumstantial, that only *Ae. vexans* selected hares as hosts in this study, and that two of the four blood meals identified at the site where TAHV-positive *Ae. vexans* were collected were hares. Given the historic association of TAHV with hedgehogs [14, 44–47], we emphasize that hedgehog blood was not detected in any mosquito in our study. Our data provide support for the hypothesis that TAHV prevalence in *Ae. vexans* populations is related to the presence and/or abundance of hares, and while larger mammals are fed upon frequently by *Ae. vexans* and have relatively high TAHV seropositive ratios, they may not be competent hosts.

Finally, we report a remarkable “stability” of the TAHV genome over time, with very few genetic changes between the two isolates and historical isolates from the region. Others have also reported similarly low genetic variation in time and over distances for *La Crosse orthobunyavirus*, another California group orthobunyavirus [84], but none has reported such low genomic variation in the California group viruses over a span of 60 years of (presumably) continuous transmission. Specifically, half of the amino acid substitutions in the G1/G2 protein and the RdRP found in the two isolates were present in other historical genomes, although as a whole there was no temporal clustering of isolates, and limited spatial clustering (the isolate from France shared a common ancestral node with isolates from the Czech Republic, Slovakia, and Austria, Fig. 3). As with other California group orthobunyaviruses, we assume that transovarial transmission (TOT) of TAHV occurs in competent TAHV vectors (*Ae. vexans*) [85, 86]. Vertical transmission allows an arbovirus to persist between seasons in a focus without an overwintering vector/host, and seasonal amplification in vertebrate hosts may not be required to maintain the virus in the environment [48]. In this case, the “stabilized infection” of the *Ae. vexans* population via vertical transmission may be assisted by occasional amplification in hares, and large mammals, while the major source of blood for *Ae. vexans*, are likely not competent hosts. A pattern of “genetic storage” has been shown for Rift Valley fever virus, where epidemic strains emerge with inter-epidemic strains when severe flooding conditions allow the hatching of large numbers of TOT-infected *Aedes* mosquitoes [87]. While such multiyear periodic flooding events are not typical in Europe, floodwater mosquitoes may delay hatching after several flooding events [88]. We hypothesize that the lack of genetic diversity in TAHV may be partially the result of “storing” high-fitness strains over years via vertical transmission, and we assume there is limited selective pressure from adaptive immunity in the vertebrate population.

These high-fitness strains likely arose during a relatively long evolutionary time between the ancestors of *Ae. vexans* and TAHV, respectively. Support for this comes from the evolutionary history of *Ae. vexans*, as the sole member of the Palaeotropical subgenus *Aedimorphus* with a Holarctic distribution. It is thought that the adaptation of *Ae. vexans* to more temperate northern climates allowed the expansion into the Nearctic during the last warm period (~ 9000 years ago) [89]. Similarly, the California serogroup viruses—and specifically the California encephalitis complex—also have a Holarctic distribution [46] with the exception of Lumbo virus (Afrotropical). The shared common ancestry of TAHV and Lumbo viruses is supported by several Bayesian phylogenetic analyses of this complex; and while it is less certain, together they may share a common ancestor with the other members of the complex diverging approximately 10,000 years ago (depending on the segment) [46, 90, 91]. The radiation of the modern California complex viruses may have followed from movement of the ancestor of *Ae. vexans* northward into the Palearctic (e.g., snowshoe hare virus, Chatanga virus) and Nearctic (La Crosse virus, Morro Bay virus), respectively.

## Conclusion

We have characterized the mosquito communities in floodplain habitats of eastern Austria. Typical floodwater mosquitoes (*Ae. vexans*, *Oc. sticticus*) were the predominant species, with increased abundance of peridomestic mosquito species at disturbed sites along the Leitha, where TAHV was isolated from two pools of *Ae. vexans*. We showed that *Ae. vexans* takes blood primarily from large vertebrates (deer and wild boar), but has a rather wide host range which includes European hares. The pattern of virus-mosquito and mosquito–host associations in this study matches the pattern of virus–host associations from a previous serosurvey performed in the region. These preliminary data should be used to begin more detailed assessments of TAHV ecology in the region, particularly the role of vertebrate hosts in the amplification of the virus and a contemporary assessment of spillover into humans. TAHV is known to infect humans and potentially causes neuropathology, although few cases are ever diagnosed and/or reported.

## Supplementary Information

**Additional file 1: Supplemental Methods**. Mosquito identification and taxonomy. **Table S1**. Published historical records of the isolation or molecular detection of *Tahyna orthobunyavirus* from mosquitoes in Europe. **Table S2**. Coordinates for approximate areas of longitudinal mosquito sampling along the Danube, Leitha, and Morava rivers in eastern Austria. **Table S3**. Sampling effort (number of trap-nights) across three floodplain habitats in Austria. **Table S4**. Primers used to amplify the complete genome of *Tahyna orthobunyavirus* (TAHV) by RT-PCR for genetic sequencing. **Table S5**. GenBank accession numbers for *Tahyna orthobunyavirus* (TAHV) isolates included in the phylogenetic analyses, listing host species/country/isolate name/year for each of three gene segments, when available (n.a. = not available). **Table S6**. Deduced amino acid changes in the viral polyproteins (G1/G2) and the polymerase (RdRP) between two *Tahyna orthobunyavirus* isolates from Austria, 2019, and when compared to the consensus sequences from Europe, 1958–1984. **Table S7**. Hosts of mosquitoes in floodplain habitats in eastern Austria identified by sequencing a portion of 16S rRNA amplified from blood in the mosquito gut. **Figure S1**. Comparison between height of the Danube river and abundance of two floodwater mosquito species at a floodplain habitat in the Donau-auen National Park, Austria, 2016.

## Data Availability

The data generated or analyzed during this study are mostly included in this published article and supplementary information files. Genetic sequences of virus isolates have been deposited in NCBI’s GenBank database (MZ245724-MZ245729). Complete datasets are available from the corresponding author on reasonable request.

## Abbreviations

TAHV: *Tahyna orthobunyavirus*
TOT: Transovarial transmission
NP: Nucleocapsid protein
G1/G2: Viral glycoprotein polyprotein precursor
RdRP: RNA-dependent RNA polymerase
