# Supplementary material for: The transmission ecology of Tahyna orthobunyavirus in Austria as revealed by longitudinal mosquito sampling and blood meal analysis in floodplain habitats
Source: Parasit Vectors. 2021 Oct 30;14:561. doi: 10.1186/s13071-021-05061-1 (PMC8556901; doi:10.1186/s13071-021-05061-1)
Supplement: Supplementary file 1 — Additional file 1: Supplemental Methods. Mosquito identification and taxonomy. Table S1. Published historical records of the isolation or molecular detection of Tahyna orthobunyavirus from mosquitoes in Europe. Table S2. Coordinates for approximate areas of longitudinal mosquito sampling along the Danube, Leitha, and Morava rivers in eastern Austria. Table S3. Sampling effort (number of trap-nights) across three floodplain habitats in Austria. Table S4. Primers used to amplify the complete genome of Tahyna orthobunyavirus (TAHV) by RT-PCR for genetic sequencing. Table S5. GenBank accession numbers for Tahyna orthobunyavirus (TAHV) isolates included in the phylogenetic analyses, listing host species/country/isolate name/year for each of three gene segments, when available (n.a. = not available). Table S6. Deduced amino acid changes in the viral polyproteins (G1/G2) and the polymerase (RdRP) between two Tahyna orthobunyavirus isolates from Austria, 2019, and when compared to the consensus sequences from Europe, 1958–1984. Table S7. Hosts of mosquitoes in floodplain habitats in eastern Austria identified by sequencing a portion of 16S rRNA amplified from blood in the mosquito gut. Figure S1. Comparison between height of the Danube river and abundance of two floodwater mosquito species at a floodplain habitat in the Donau-auen National Park, Austria, 2016. [file 13071_2021_5061_MOESM1_ESM.docx]

**Supplemental Methods: mosquito identification and taxonomy**. We did not differentiate between morphologically similar species/species complexes. We use “*sensu lato*” (s.l.) when referring to *Anopheles maculipennis* s.l., as this may be any member of this complex, e.g., *Anopheles maculipennis* (Meigen 1818) or *Anopheles messeae* Falleroni 1926. We use the same convention for *Culex pipiens* s.l., which may refer to both *Culex pipiens pipiens* Linnaeus 1758 and the morphologically similar *Culex torrentium* Martini 1925*.* Previous molecular barcoding by our lab and others (see Werblow, et al. 2016. *Med Vet Entomol* 30:8-13) suggested to us that we encountered *Ochlerotatus cantans* (Meigen 1818) and not *Ochlerotatus annulipes* (Meigen 1830) (both are likely *incertae sedis* with respect to subgenus). Similarly, we also believe we encountered *Aedes* (*Aedes*) *cinereus* (Meigen 1818) and not *Aedes* (*Aedes*) *geminus* Peus 1970. A complete taxonomic list of encountered species is as follows, sorted by Tribe, Genus, and Subgenus:

Culicinae: Aedini

*Aedes* (*Aedes*) *cinereus* (Meigen 1818)

*Aedes* (*Aedimorphus*) *vexans* (Meigen 1830)

*Aedes* (*Dahliana*) *geniculatus* (Olivier 1791)

*Ochlerotatus* *cantans* (Meigen 1818)

*Ochlerotatus* (*Ochlerotatus*) *sticticus* (Meigen 1838)

Culicinae: Culicini

*Culex* (*Baraudius*) *modestus* Ficalbi 1890

*Culex* (*Culex*) *pipiens* *pipiens* Linnaeus 1758

*Culex* (*Neoculex*) *territans* (Walker 1856)

*Cules* (*Culex*) *torrentium* Martini 1925

Culicinae: Culisetini

*Culiseta* (*Allotheobaldia*) *longiareolata* (Macquart 1838)

*Culiseta* (*Culiseta*) *annulata* (Schrank 1776)

Culicinae: Mansoniini

*Coquillettidia* (*Coquillettidia*) *richiardii* (Ficalbi 1889)

Anophelinae

*Anopheles* (*Anopheles*) *claviger* (Meigen 1804)

*Anopheles* (*Anopheles*) *hyrcanus* (Pallas 1771)

*Anopheles* (*Anopheles*) *maculipennis* Meigen 1818

*Anopheles* (*Anopheles*) *messeae* Falleroni 1926

*Anopheles* (*Anopheles*) *plumbeus* Stephens 1828

**Table S1**. Published historical records of the isolation or molecular detection of Tahyna orthobunyavirus from mosquitoes in Europe.

| **Country** | **Species** | **Year(s)** | **Method** | **Reference** |
| --- | --- | --- | --- | --- |
| Slovakia | *Aedes vexans*  *Ae. caspius* | 1958 | isolation | a. |
| France | mosquitoes | 1966 | isolation | b. |
| Austria | *Ae. vexans* | 1966 | isolation | c. |
| Italy | *Aedes* spp | 1968 | isolation | d. |
| Romania | *Culex pipiens* | 1971 | isolation | e. |
| Czech Republic | *Ae. vexans*  *Ae. cantans*  *Ae. cinereus*  *Ae. caspius* | 1972 | isolation | f. |
| Slovakia | *Culiseta annulata* (larvae) | 1974 | isolation | g. |
| Czech Republic | *Ochlerotatus. sticticus*  *Cx. modestus* | 1975 | isolation | h. |
| Czech Republic | *Ae. cinereus*  *Ae. vexans* | 1997 | isolation | i. |
| Russia | *Ae. communis*  *Ae. excrucians* | 1998 | isolation | j. |
| Czech Republic | *Ae. cinereus*  *Ae. vexans* | 1999 | isolation | k. |
| Czech Republic | *Ae. cinereus*  *Oc. sticticus*  *Ae. vexans*  *Cx. modestus* | 2006 | isolation | l. |
| Italy | *Ae. caspius* | 2008 | detection | m. |
| Austria | *Cx. pipiens*/*torrentium* | 2011 | detection | o. |
| Serbia | *Ae. vexans* | <1976 | isolation | p. |
| Slovakia | *Ae. vexans* | 1973, 1975 | isolation | p. |
| Germany | *Ae. vexans* | 1979-1981 | isolation | q. |
| Austria | *Ae. caspius* | 1979-1981 | isolation | q. |
| Germany | *Ae. vexans* | 1995-1997 | detection | r. |
| Italy | *Ae. caspius*  *Ae. vexans* | 2009-2010 | detection | s. |
| Czech Republic | *Anopheles hyrcanus* | 2013 | isolation | t. |
| Austria | *Ae. vexans* | 2019 | isolation | u |

a. Bardos V, Danielova V. (1959) The Tahyna virus - a virus isolated from mosquitoes in Czechoslovakia 3:264-276

b. Hannoun C, Panthier R, Corniou B. (1966) Isolation of Tahyna virus in the south of France. Acta Virol. 10(4):362-364.

c. Aspöck H, Kunz C (1966) [Isolation of the Tahyna virus from mosquitoes in Austria]. Archiv für die gesamte Virusforschung 18:8-15.

d. Balducci M, Verani P, Lopes MC, Saccà G, Gregorig B. Isolation of Tahyna virus from *Aedes* mosquitoes in Northern Italy (Gorizia Province). Acta Virol. 1968 Sep;12(5):457-459.

e. Arcan P, Topciu V, Rosiu N, Csaky N. (1974) Isolation of Tahyna virus from *Culex pipiens* mosquitoes in Romania. Acta virologica 18(2):175

f. Danielova V, Malkova D, Minar J, Ryba J. (1976) Dynamics of the natural focus of Tahyna virus in southern Moravia and species succession of its vectors, the mosquitoes of the genus *Aedes*. Folia parasitologica 23(3):213-219

g. Bardos V. Ryba J, Hubalek Z, Olejnicek J (1978) Virological examination of mosquito larvae from southern Moravia. Folia Parastiologia 25(1):75-78

h. Danielova V, Holubova J. (1977) Two more mosquito species proved as vectors of Tahyna virus in Czechoslovakia. Folia Parasitologia 24(2):187–189

i. Hubálek Z, Savage HM, Halouzka Z. Sanogo YO, Lusk S. (2000) West Nile virus investigations in South Moravia Czechland. Viral Immunol. 13(4):427-433

j. L’vov DK, Gromashevskii VL, Skvortsova TM, Aristova VA. Kolobukhina LV, Morozova TN, Galkina IV, Butenko AM, Nedialkova MS, Selivanov IaM. et al (1998) [Circulation of viruses of the California serocomplex (Bunyaviridae, Bunyavirus) in the central and southern parts of the Russian plain]. Voprosy Virusologii 43(1):10–14

k. Hubálek Z, Halouzka J, Juricová Z, Príkazský Z, Záková J, Sebesta O. (1999) [Surveillance of mosquito-borne viruses in Breclav after the flood of 1997]. Epidemiol Mikrobiol Imunol. 48(3):91-6.

l. Hubálek Z, Rudolf I, Bakonyi T, Kazdová K, Halouzka J, Sebesta O, Sikutová S, Juricová Z, Nowotny N. (2010) Mosquito (Diptera: Culicidae) surveillance for arboviruses in an area endemic for West Nile (Lineage Rabensburg) and Tahyna viruses in central Europe. J Med Entomol 47(3):466-472

m. Calzolari M, Bonilauri P, Bellini R, Caimi M, Defilippo F, et al. (2010) Arboviral survey of mosquitoes in two northern Italian regions in 2007 and 2008. Vector-borne and Zoonotic Diseases 10(9):875-884

n. Sonnleitner ST, Lundström J, Baumgartner R, Simeoni J, Schennach H, Zelger R, Prader A, Schmutzhard E, Nowotny N, Walder G. (2014) Investigations on California serogroup orthobunyaviruses in the Tyrols: first description of Tahyna virus in the Alps. Vector-borne and Zoonotic Diseases 14(4):272-277

o. Gligic A, Adamovic ZR. (1976) Isolation of Tahyna virus from *Aedes vexans* mosquitoes in Serbia. Mikrobiologija 13(2):119–129

p. Danielova V, Malkova D, Minar J, Rehse-Küpper B, Hajkova Z, Halgos J, Jedlicka L. (1978) Arbovirus isolations from mosquitoes in south Slovakia. Folia Parasitologia 25(2):187–191

q. Pilaski J, Mackenstein H. (1985) Isolation of Tahyna virus from mosquitoes in 2 different European natural foci. Zbl. Bakt.Mikro.Hyg.B. 180(4):394-420

r. Schüssler E (2000) Tahyna-Virus: Untersuchungen zum vorkommen am Oberrhein und sequenzvergleiche des M-Segments bei zehn Virusisolaten. Thesis, Ruprecht-Karls-Universität Heidelberg, Germany, 140pp

s. unpublished GenBank sequences, Bonilauri P, Calzolari M, Barbieri I. Tahyna virus activity detected in field collected *Aedes* mosquitoes in Italy. Submitted 07-Apr-2010

t. Hubálek Z, Sebesta O, Pesko J, Betasova L, Blazejova H, enclikova K, Rudolf I (2014) Isolation of Tahyna virus (California encephalitis group) from *Anopheles hyrcanus* (Diptera, Culicidae), a mosquito species new to, and expanding in, Central Europe. J Med Entomol 51(6):1264-1267

u. this study

**Table S2**. Coordinates for approximate areas of longitudinal mosquito sampling along the Danube, Leitha, and Morava rivers in eastern Austria.

| **Floodplain** | **Site name** | **Latitude** | **Longitude** |
| --- | --- | --- | --- |
| Danube | Schönau | 48.13°N | 16.61°E |
|  | Orth | 48.12°N | 16.70°E |
|  | Eckartsau | 48.12°N | 16.78°E |
|  | Witzelsdorf | 48.13°N | 16.84°E |
| Morava | Marchegg1 | 48.29°N | 16.89°E |
|  | Marchegg2 | 48.30°N | 16.89°E |
|  | Hohenau | 48.60°N | 16.93°E |
| Leitha | Bruck1 | 48.01°N | 16.75°E |
|  | Bruck2 | 48.02°N | 16.80°E |
|  | Rohrau | 48.06°N | 16.86°E |

**Table S3**. Sampling effort (number of Trap-nights) across three floodplain habitats in Austria

|  |  | **Month** | | | | |  |
| --- | --- | --- | --- | --- | --- | --- | --- |
| **Floodplain** | **Year** | **May** | **June** | **July** | **August** | **September** | **Total** |
| Danube | 2016 | 2 | 5 | 10 | 4 | 2 | **23** |
|  | 2017 | 1 | 5 | 3 | 2 |  | **11** |
| Morava | 2016 |  |  | 3 |  |  | **3** |
|  | 2017 |  | 1 | 4 | 2 | 1 | **8** |
| Leitha | 2017 |  | 2 | 2 | 1 |  | **5** |
|  | 2019 |  | 10 | 13 | 11 |  | **34** |
| *Total* |  | *3* | *23* | *35* | *20* | *3* | ***84*** |

**Table S4**. Primers used to amplify the complete genome of *Tahyna orthobunyavirus* (TAHV) by RT-PCR for genetic sequencing.

| **PCR** | **Primer** | **Sequence** | **Reference** |
| --- | --- | --- | --- |
| 1 | TahS20f | GAGATACCGAGAGGAATAAC | a |
|  | TahS692r | CTCTTAGCTATGGCAGTAGT | a |
| 2 | TahS226f | AAGCTGCTCTCGCTCGTAAG | b |
|  | TahS972r | GTGTGCTCCACTGAATACCT | b |
| 3 | TahM5pF | AGTAGTGTACTACCAAGTATAGATAA | c |
|  | TahM616R | TGTAAGAATCTAACACAAGACATGT | c |
| 4 | TahM125f | CAGGATGGTGCAATCGTCAA | a |
|  | TahM1117r | CATTGCCATGCTCAGCATGT | a |
| 5 | TahM508F | TATCACCCTTGATCAAACTTGCG | c |
|  | TahM1359R | TGCACGAGGCAGTTGTATGTTTT | c |
| 6 | TahM883f | CGAGACATCGGATAGGATGA | a |
|  | TahM1394r | GCACGAGGCAGTTGTATGTT | a |
| 7 | TahM1250f | TGCCACGAGTGCGATATGTA | a |
|  | TahM2008r | TGCGTGGTGTAAGGACATCA | a |
| 8 | TahM1921f | CGCCAATGAGATGAAGAACT | a |
|  | TahM2888r | GGCTTGTCTGCAACTTCTGT | a |
| 9 | TahM2786f | ACGTGGCAGGTTAGTCACAA | a |
|  | TahM3510r | CGCACTGCAATGATTCTAGG | a |
| 10 | TahM3478f | GGCTTACAGTCTGCCTAGAA | a |
|  | TahM4102r | CTTAGCATCTGCCAGTGTCA | a |
| 11 | TahM3794f | AAGGCTATCCTTGGAGATGT | a |
|  | TahM4408r | CCAATTCCTTAGCAGCTATG | a |
| 12 | TahL152F | ATGGCCAGACATGATTATTTTG | d,e |
|  | TahL768R | CCACCTTTCAGATTCATAAGC | d,e |
| 13 | TahL637F | ATGGTGTAAAGAAGGATGTCC | d,e |
|  | TahL1465R | GCTTTTGGACAATATCTTCT | d,e |
| 14 | TahL1316F | TCTGCGGAATAGGGAAGCATAA | d,e |
|  | TahL1725f | TAGTATCGTGGTCTTGCACA | a |
| 15 | TahL2409r | TGTGGACCATATTTCAGTTA | a |
|  | TahL2248R | CTGGAAACCATATGCTAACTAA | d,e |
| 16 | TahL3236F | AGATATGTCTAAATGGAGTGCTCA | d,e |
|  | TahL4197R | GCTATTTGGTTGACTACTGA | d,e |
| 17 | TahL2218F | AGGTATAAAAGACAACAGAGAAT | d,e |
|  | TahL3295R | GGCTATGAGCCAGAAGTATT | d,e |
| 18 | TahL3551F | AGCTGTGCAATGTCAGTATATAAG | d,e |
|  | TahL4620R | TCTATTATGGGCTTATGTGA | d,e |
| 19 | TahL4425F | CCTGGTGGAATGGAGGAGCTATTT | d,e |
|  | TahL5393R | ATGAGTCTGCAAAATGGGTAA | d,e |
| 20 | TahL5205F | GCATATACGACAACTGACTTCTGCG | d,e |
|  | TahL6402R | CTGTCTATTAGTTGCTTTATTGC | d,e |

a. In house

b. Hubálek et al. 2010. “Mosquito (Diptera: Culicidae) surveillance for arboviruses in an area endemic for West Nile (Lineage Rabensburg) and Tahyna viruses in Central Europe”. *J Med Entomol* 47(3):466472

c. Kilian et al. 2010. “Nucleotide variability of Tahyna virus (Bunyaviridae, Orthobunyavirus) small (S) and medium (M) genomic segments in field strains differing in biological properties” *Vir Research* 149(1):119-123.

d. Vanlandingham, et al. 2002. “Molecular characterization of California serogroup viruses isolated in Russia.” *Am J Trop Med Hyg* 67(3): 306-309.

e. Kilian et al. 2013. “The variability of the large genomic segment of Tahyna orthobunyavirus and an all-atom exploration of its anti-viral drug resistance.” *Infect Gene Evolution* 20:304-311.

**Table S5**. GenBank accession numbers for *Tahyna orthobunyavirus* (TAHV) isolates included in the phylogenetic analyses, listing host species/country/isolate name/year for each of three gene segments, when available (n.a. = not available).

| **TAHV isolate** | **S segment** | **M segment** | **L segment** |
| --- | --- | --- | --- |
| *Aedes vexans*/Austria/OadD806/2019 | MZ245724 | MZ245726 | MZ245728^a^ |
| *Aedes vexans*/Austria/OadD823/2019 | MZ245725 | MZ245727 | MZ245729 |
| *Aedes vexans*/Czech_Republic/5060/1968 | GU390688 | GQ386841 | n.a. |
| *Aedes vexans*/Czech_Republic/4060/1966 | GU390687 | GQ386840 | n.a. |
| *Aedes vexans*/Czech_Republic/4057/1966 | GU390686 | GQ386839 | KF361884 |
| *Aedes vexans*/Czech_Republic/4034/1966 | GU390685 | GQ386838 | KF361883 |
| *Aedes vexans*/Czech_Republic/4033/1966 | GU390684 | GQ386837 | n.a. |
| *Aedes vexans*/Czech_Republic/4030/1966 | GU390683 | GQ386836 | n.a. |
| *Aedes vexans*/Czech_Republic/4020/1966 | GU390682 | GQ386835 | KF361882 |
| *Aedes vexans*/Czech_Republic/4019/1966 | GU390681 | GQ386834 | n.a. |
| *Aedes vexans*/Czech_Republic/1014/1964 | GU390680 | GQ386833 | KF361881 |
| *Aedes vexans*/Czech_Republic/181/1962 | GU390678 | GQ386831 | KF361878 |
| *Aedes vexans*/Czech_Republic/106/1963 | GU390676 | GQ386830 | n.a. |
| *Aedes vexans*/Czech_Republic/105/1963 | GU390675 | GQ386829 | n.a. |
| *Aedes cantans*/Czech_Republic/94/1963 | GU390674 | GQ386828 | KF361877 |
| *Aedes vexans*/Czech_Republic/90/1963 | GU390673 | GQ386827 | n.a. |
| *Aedes vexans*/Czech_Republic/89/1963 | GU390672 | GQ386826 | KF361876 |
| *Aedes vexans*/Czech_Republic/83/1963 | GU390671 | GQ386825 | n.a. |
| *Aedes vexans*/Czech_Republic/76/1963 | GU390670 | GQ386824 | KF361875 |
| *Aedes vexans*/Czech_Republic/29/1963 | GU390669 | GQ386823 | KF361874 |
| n.a./"Czechoslovakia"/22595-6/1984^b^ | HM036217 | HM036218 | HM036219 |
| n.a./France/Souche_D_C14019-29/1968 | HM036214 | HM036215 | HM036216 |
| *Aedes caspius*/Slovakia/92Bardos/1958 | HM036208 | HM036212 | HM036210 |
| *Aedes vexans*/Slovakia/236/1958 | GU390679 | GQ386832 | KF361880 |
| *Aedes dorsalis*/China/NM08003/2008 | HQ541823 | n.a. | n.a. |
| *Aedes vexans*/China/XJ0710/2007 | HM243142 | HM243141 | HM243140 |
| *Aedes vexans*/China/XJ0708/2007 | HM243139 | HM243138 | HM243137 |
| *Culex* sp./China/XJ0625/2006 | EU622820 | EU622819 | EU665255 |

a. sequencing of the L segment was incomplete for isolate OadD806, missing 858 nucleotides between 2357-3215. b. specific location information is not available.

**Table S6.** Deduced amino acid changes in the viral polyproteins (G1/G2) and the polymerase (RdRP) between two Tahyna orthobunyavirus isolates from Austria, 2019, and when compared to the consensus sequences from Europe, 1958-1984.

| **Gene (number of sequences compared)** | **OadD806** | **OadD823** | **Number of isolates with shared substitutions** |
| --- | --- | --- | --- |
| G1/G2 (*N* = 24) |  | M362I | 2 |
|  | K504M |  | 0 |
|  | I528M |  | 1 |
|  |  | E536D | 0 |
|  |  | E668G | 2 |
|  |  | R859K | 2 |
|  | T1034I |  | 0 |
| RdRP (*N* = 15) |  | S141N | 0 |
|  |  | R345K | 0 |
|  | Y451F |  | 3 |
|  |  | V630I | 1 |
|  | N775S |  | 0 |
|  |  | V1486I | 3 |
|  | V2191 | I2191 | V2191 = 7,  I2191 = 8 |
|  | K2227M |  | 0 |

**Table S7.** Hosts of mosquitoes in floodplain habitats in eastern Austria identified by sequencing a portion of 16S rRNA amplified from blood in the mosquito gut.

| **Mosquito** | ***Cervus elaphus*** | ***Capreolus capreolus*** | ***Sus scrofa*** | ***Homo sapiens*** | ***Lepus europaeus*** | ***Bos taurus*** | ***Castor fiber*** | ***Pelophylax* sp.^d^** | ***Equus caballus*** | ***Felis silvestris*** | ***Hyla arborea*** | ***Meles meles*** | **negative** | **Total** |
| --- | --- | --- | --- | --- | --- | --- | --- | --- | --- | --- | --- | --- | --- | --- |
| *Ae. vexans* | 62 | 36^c^ | 20^c^ | 1 | 4 | 2 |  |  | 1 | 1 |  | 1 | 13 | 141 |
| *Oc. sticticus* | 30^ab^ | 10^a^ | 27 | 5^b^ |  |  | 2 | 1 |  |  |  |  | 7 | 82 |
| *Ae. cinereus* | 1^a^ | 5^ac^ | 1c |  |  |  |  |  |  |  |  |  | 5 | 12 |
| *Cq. richiardii* |  | 2 | 3 |  |  |  |  |  |  |  |  |  | 1 | 6 |
| *An. maculipennis* s.l. | 1 | 1 |  |  |  |  |  |  |  |  |  |  | 2 | 4 |
| *Cx. territans* |  |  |  |  |  |  |  | 1 |  |  | 1 |  | 1 | 3 |
| *Oc. annulipes* |  | 1 |  |  |  |  |  |  |  |  |  |  | 1 | 2 |
| *Cx. pipiens* s.l. |  |  |  |  |  |  |  |  |  |  |  |  | 28 | 28 |
| **Total** | **94** | **55** | **51** | **6** | **4** | **2** | **2** | **2** | **1** | **1** | **1** | **1** | **58** | **278** |

Mixed blood meals were counted twice: a. includes one mixed blood meal from *Cervus elaphus* and *Capreolus capreolus* (*Oc. sticticus* = 1, *Ae. cinereus* = 1); b. includes one mixed blood meal from *Cervus elaphus* and *Homo sapiens*; c. includes mixed bloodmeals from *Sus scrofa* and *Capreolus capreolus* (*Ae. vexans* = 2, *Ae. cinereus* = 1).


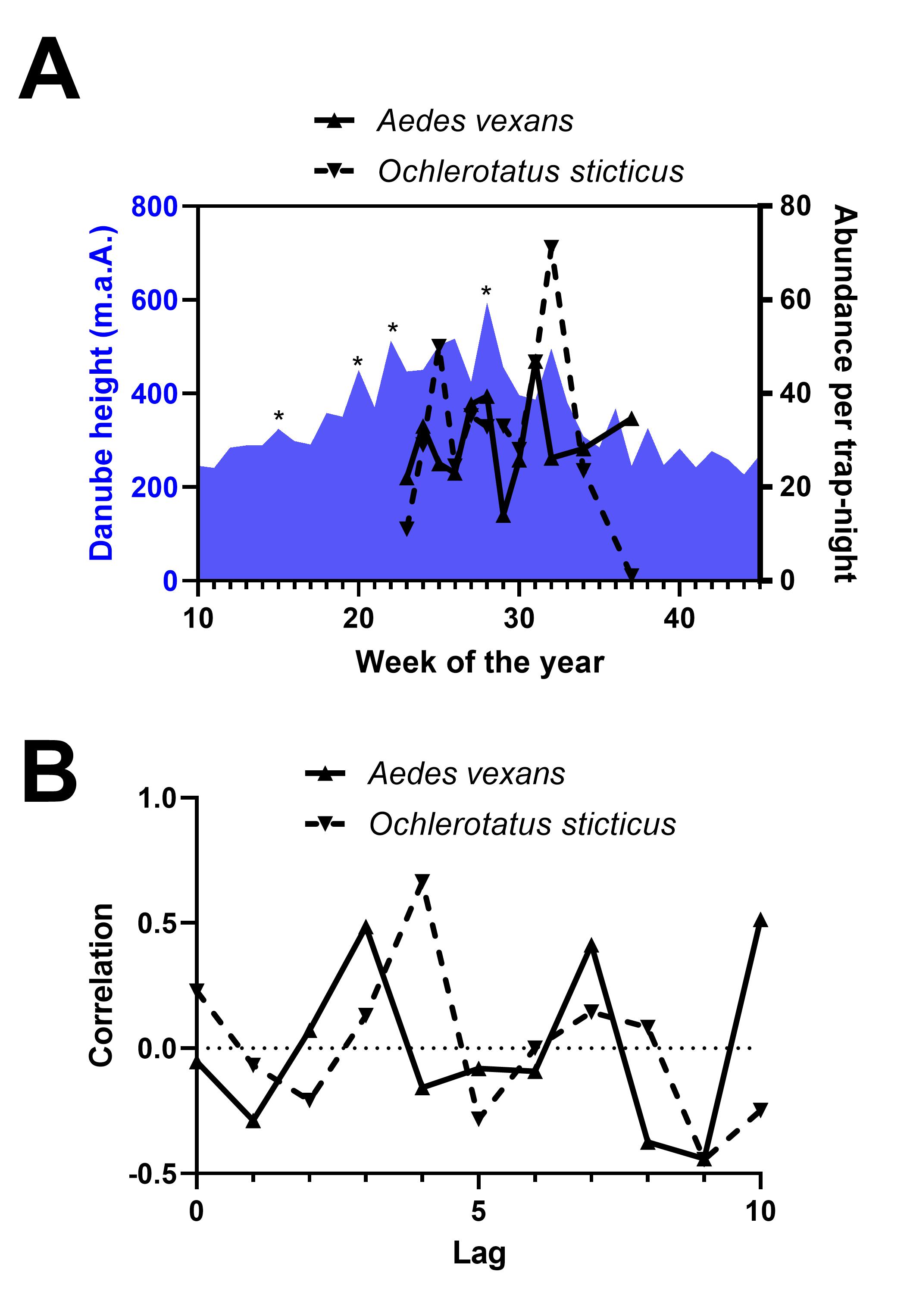


**Figure S1**. Comparison between height of the Danube river and abundance of two floodwater mosquito species at a floodplain habitat in the Donau-auen National Park, Austria, 2016. (A) The maximum weekly water height (blue, in meters above Adriatic, “m.a.A.”) was measured from a point up-river from the collection sites, accessed from publicly available data of the Austrian Ministry for Agriculture, Regions, and Tourism (<https://wasser.umweltbundesamt.at/hydjb/search/search.xhtml>). Asterisks indicate monthly high-water (presumed flooding events). Mosquito abundances (*Aedes vexans*, solid line; *Ochlerotatus sticticus*, dashed line) were counts per trap night, captured weekly using CDC light traps with CO_2_ from May-September, 2016. (B) The Pearson product-moment correlation was calculated between abundance and maximum water height for each week over 10 weeks when sampling was performed, and then lagged by 1 week intervals to show maximum correlation for *Ae. vexans* at 3 week lag and *Oc. sticticus* at 4 week lag.


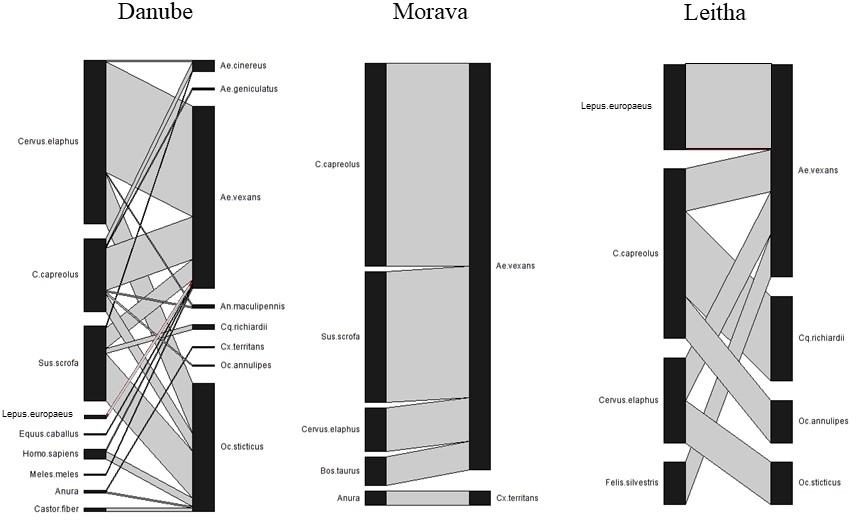


**Figure S2.** Bipartite plot of the hosts (left) of mosquitoes (right) sampled from habitats along three floodplains in eastern Austria: the Danube river, the Morava river, and the Leitha river. The size of the black bars indicates the relative abundance of each species within a given floodplain, but the scales are different for each floodplain. For reference, the bars represent 92 individual *Aedes vexans* along the Danube river, 28 along the Morava river, and 4 along the Leitha river.
